# Lignocellulosic Bionanomaterials for Biosensor Applications

**DOI:** 10.3390/mi14071450

**Published:** 2023-07-19

**Authors:** Ekrem Durmaz, Selva Sertkaya, Hande Yilmaz, Cagri Olgun, Orhan Ozcelik, Ayhan Tozluoglu, Zeki Candan

**Affiliations:** 1 Department of Forest Industrial Engineering, Kastamonu University, 37200 Kastamonu, Turkey; edurmaz@kastamonu.edu.tr (E.D.);; 2 Department of Forest Industrial Engineering, Duzce University, 81620 Duzce, Turkey; selvasertkaya@duzce.edu.tr (S.S.);; 3 Department of Aerospace Engineering, Ankara Yildirim Beyazit University, 06010 Ankara, Turkey; 4 Biomaterials and Nanotechnology Research Group & BioNanoTeam, 34473 Istanbul, Turkey; 5 Department of Forest Industrial Engineering, Istanbul University Cerrahpasa, 34473 Istanbul, Turkey

**Keywords:** lignocellulosic biomass, nanotechnology, bionanomaterials, nanocellulose, nanolignin, biosensor

## Abstract

The rapid population growth, increasing global energy demand, climate change, and excessive use of fossil fuels have adversely affected environmental management and sustainability. Furthermore, the requirements for a safer ecology and environment have necessitated the use of renewable materials, thereby solving the problem of sustainability of resources. In this perspective, lignocellulosic biomass is an attractive natural resource because of its abundance, renewability, recyclability, and low cost. The ever-increasing developments in nanotechnology have opened up new vistas in sensor fabrication such as biosensor design for electronics, communication, automobile, optical products, packaging, textile, biomedical, and tissue engineering. Due to their outstanding properties such as biodegradability, biocompatibility, non-toxicity, improved electrical and thermal conductivity, high physical and mechanical properties, high surface area and catalytic activity, lignocellulosic bionanomaterials including nanocellulose and nanolignin emerge as very promising raw materials to be used in the development of high-impact biosensors. In this article, the use of lignocellulosic bionanomaterials in biosensor applications is reviewed and major challenges and opportunities are identified.

## 1. Introduction

The aim of generating an eco-friendly and sustainable world has gradually increased the request for renewable bio-based natural sources on a global scale. Non-renewable fossil resources are not sustainable as well as they have negative environmental influences. For this reason, environmentally-friendly alternative resources have long been sought to replace the fossil [1]. Bio-based raw materials such as forest and agricultural wastes, wood, crops, and food residues have been widely considered as appropriate benign renewable resources that can be utilized to manufacture new generation value-added materials. The plants consist of lignocellulosic structures involving cellulose (40–50%), hemicellulose (20–40%) and lignin (20–30%) are known to be the most common and accessible bio-resources on the planet [2].

Being the most abundant natural polymer on earth with an annual production of about 10^11^–10^12^ tons, cellulose has a great value and potential for obtaining eco-friendly bio-based products [3]. Coniferous and deciduous trees, annual plants such as bamboo, bagasse, raphia palm, etc., and also most agricultural residues and sea creatures known as tunicate as well as some bacteria and fungi can be considered cellulose sources [4]. In 1838, Payen was the first to identify a major insoluble waste called cellulose. Since its discovery, thousands of scientific papers, patents, and books have been published concerning the importance of this natural polymer [5]. Cellulose (C_6_H_10_O_5_)_n_ is a high molecular weight homopolysaccharide constituted of β-1,4-anhydro-d-glucopyranose units (AGUs), and the glucose rings rotated through an angle of 180° about the molecular axis and hydroxyl groups in an equatorial position [6]. In this way, the two neighboring glucose rings form the auxiliary unit called cellobiose [7]. It is packed into microfibrils that are kept together by intra- and inter-molecular hydrogen bonds as well as intermolecular van der Waals forces with both non-reducing and reducing ends [8]. These intra- and inter-chain non-covalent attractions are important for the stability and firm structure of cellulose, the latter of which is basic for plants and some marine creatures [9]. Furthermore, the two ends of cellulose, each AGU has one primary hydroxyl group on C6 and two secondary hydroxyl groups on C2 and C3. A large number of hydroxyl groups make cellulose molecules easy to compose intermolecular or intramolecular hydrogen bonds. It is noteworthy that the 1HO(2) and 1HO(6) hydroxyl groups of the forming glucose units act as hydrogen-bond donors to water, whereas the 1HO(3) groups behave exclusively as hydrogen-bond acceptors from water and donate hydrogen to their intra-chain neighbors O(5) [10,11]. The presence of the strong hydrogen bonding clarifies it with the crystalline nature, rigidity, and un-reactiveness toward water and other chemicals [12]. On the one hand, the weak hydrogen bonds present in amorphous zones demonstrates hydrophilicity, accessibility, and flexibility. The structure of cellulose establishes the features such as degradability, hydrophilicity, chemical variability, and chirality owing to the presence of OH groups [13].

The essential drawback of the pristine cellulose is that it is difficult to process owing to the fact that strong intramolecular and intermolecular interactions make it very difficult to dissolve in typical organic solvents. Due to their outstanding dissolving capabilities, cellulose derivatives are potential alternatives to pristine cellulose. Ethanolic or aqueous hydrolysis can also be utilized to transform them to cellulose. Cellulose ethers (methyl cellulose, ethyl cellulose, and carboxymethyl cellulose), cellulose esters (cellulose acetate), cellulose nitrate, and cellulose sulfate are the most well-known cellulose derivatives [14]. Cellulose acetate is a renewable material that has gradually attracted considerable research interest because of its potential benefits such as being biocompatible, non-toxic, non-corrosive, and biodegradable. It is usually used as a dispersant to equally distribute nanoparticles in a suspension [15].

Lignin is a three-dimensional and quite complex phenolic chemical compound and, after cellulose, is the most widely available polymer in the earth [16,17,18,19]. Due to its complex structure, lignin occurs in significant quantities as a by-product of the pulp industry and bioethanol manufacturing processes, but only a few of them are currently used at an industrial level [16,20,21,22,23]. The characteristics of lignin show changes based on the type of lignocellulosic raw material obtained, such as softwood, hardwood, or annual plant [24]. Furthermore, pulping conditions, isolation methods, and other procedures applied to lignocellulosic raw materials will provide lignin with different properties and structures. This diversity and complexity directly affect the usability of lignin in many potential applications. However, regardless of the source and production method, all types of lignin have chemical functional groups that are adaptable to modifications for many different purposes, such as aliphatic and aromatic hydroxyl, carbonyl, methoxyl, as well as phenyl groups [17,25].

## 2. Nanocellulose

Nanotechnology has now established itself as being one of the most significant drivers of a current industrial revolution in many different fields of study and industries such as electronics, food, pulp and paper, pharmaceuticals, plastics, automotive, cosmetics, etc. [26,27]. Cellulose is one of the most important chemical components which constitute the structure of lignocellulosic biomass and it has found a very wide usage area in the industries such as pulp and paper production, packaging, textile products, and food additives. Nevertheless, over the last 15–20 years cellulose has gone beyond its conventional use with the discovery of nanocellulose, and with the development of nanotechnology, nanocellulose has attracted increasing attention from groves of academe and different branches of the industry [28,29]. Nanocellulose or nano-structured cellulose is the general name of all forms of cellulose-based nanoparticles which have at least one dimension less than 100 nm [30,31]. Nanocellulose has some extraordinary properties of a high degree of polymerization, high crystallinity, low density, dimensional stability, large aspect ratio, high tensile strength, high stiffness, high Young’s modulus, high specific surface area, lightweight, high transparency, enhanced thermal stability, biodegradability, biocompatibility, non-toxicity, and moldability into a three-dimensional structure [30,32,33,34,35]. Thanks to the excellent features mentioned above, nanocellulose has found extensive use in different industrial applications such as paper and paperboard production, the packaging industry, energy and electronic devices, optical products, biomedical materials, tissue engineering, adsorbent for environmental remediation, pickering emulsions, reinforcing fillers, cosmetic formulations as well as coating and varnish formulations [36,37,38].

Generally, nanocellulose is classified into two base groups: cellulose nanofibril (CNF) and cellulose nanocrystal (CNC) [39,40,41]. However, bacterial nanocellulose (BNC) has been included in the classification as a third group by many researchers [42,43,44,45]. Both CNF and CNC are obtained via top-down production processes [13,46], while BNC is achieved via a bottom-up approach in which cellulose nanofibers are secreted extracellularly by some bacteria [47]. Although the chemical structure of these three groups of nanocellulose is quite similar, morphology, dimension, and other properties indicate some important differences that depend on raw material, method of synthesis, and conditions of production processes [35]. The production of nanocellulose from cellulose contains two steps: (1) removal of non-cellulosic components which are hemicellulose and lignin via acid chlorite or alkaline pre-treatments followed by (2) obtaining nanocellulose from cellulose fibrils through different mechanical, chemical or enzymatic extraction processes [48].

Cellulose nanofibril (CNF), which is also termed as cellulose nanofiber [49], nanofibrillar cellulose [50] or nanofibrillated cellulose [51], is long, flexible and entangled spaghetti-like nanocellulose type that is a few micrometers in length, 5–60 nm in diameter with very high aspect ratio and contain not only crystalline both also amorphous domains of cellulose [31,37,52]. CNF is produced via numerous mechanical treatments such as high-pressure homogenization, microfluidization, grinding, cryo-crushing, ultrasonication, steam explosion, refining, blending, extrusion, ball milling, aqueous counter collision, and electrospinning. Isolation of CNF by applying only mechanical treatments has necessitated high energy costs. Therefore, the recent research efforts have focused on improving fibrillation and reducing energy consumption. The current processes for CNF production contain different biological and chemical pretreatments. On the one hand, these pretreatments significantly influence the features of obtained CNF. In the literature, it has been mentioned that various pretreatments such as TEMPO-oxidation, enzymatic hydrolysis, quaternization, carboxymethylation, carboxylation, sulphonation, deep eutectic solvents, and ionic liquid treatment have been conducted for the production of CNFs [3,38,49,53].

Cellulose nanocrystal (CNC), which is also referred to as crystalline nanocellulose [54], nanocrystalline cellulose (NCC) [55], or cellulose nanowhisker (CNW) [56], is synthesized via acid hydrolysis treatment from various cellulosic biomass. CNC, which has the only crystalline zones of cellulose, is a high-purity whisker with a rod-like shape as having a high aspect ratio with diameters less than 100 nm and lengths varying between 100 and 1000 nm [57,58,59]. The traditional process for the isolation of CNC is inorganic strong acid hydrolysis, such as sulfuric acid, hydrochloric acid, nitric acid, hydrobromic acid, and phosphoric acid. Among these types of acids, sulfuric acid hydrolysis is generally accepted as a well-known and effective treatment for producing CNC with good dispersibility in water. Despite the fact that strong acid hydrolysis in CNC production is regarded as a simple and timesaver treatment, some issues such as serious environmental pollution, comparatively low acquiring yield, corrosion in the equipment, and abundant water utilization need to be addressed. On the one hand, a few environmentally benign processes related to renewable and recyclable chemicals such as enzymatic hydrolysis, organic acid (e.g., oxalic acid, formic acid) hydrolysis, solid acid (e.g., phosphotungstic acid) hydrolysis, deep eutectic solvents treatment, oxidation degradation or ionic liquid have been suggested recently to overcome the aforementioned problems [38,53,60,61].

In the production of CNF and CNC, wood and wood-based biomass or materials (e.g., aspen wood, balsa, wood powder, sulfite pulp, bleached sulfite pulp, and bleached kraft pulp), various annual plants, agricultural plants and their wastes (e.g., wheat straw, sunflower stalk, rice husk, corncob residue, oil palm empty fruit bunch, cotton, sugar palm fiber, ramie, sugarcane bagasse, areca nut husk, bamboo, rice straw, pomelo peel, jute, lemongrass, lemon seed, ginger, mulberry, water hyacinth, barley straw, kenaf, corn husk, banana rachis, kiwi pruning waste, cucumber peel, chili fiber, hemp, oat husk, pomelo fruit fiber, cattail, garlic straw residue, coconut husk, banana peel, soy hull, sisal, coir, potato residue, pineapple peel, elephant grass, mengkuang leaf, mango seed, coffee husk, onion skin, pistachio shell), and also tunicate which is a marine invertebrate animal (specifically, the member of *subphylum Tunicata*) have been used [13,32,62,63,64,65].

Bacterial nanocellulose (BNC), which is also named microbial cellulose or bio-nanocellulose, is extracted from the fermentation of glucose or other carbohydrate resources through bacterial pathways. The diameter of BNC changes between 20 and 100 nm arranged in different types of nanofiber networks [52,66]. In the obtaining of BNC, different types of bacteria such as *Achromobacter, Escherichia, Zooglea*, *Salmonella, Rhizobium*, *Sarcina ventriculi*, *Azotobacter*, *Lactobacillus mali*, *Alcaligenes*, *Aerobacter*, *Pseudomonas*, *Acetobacter xylinum*, *Acanthamoeba*, *Gluconacetobacter xylinus*, *Gluconacetobacter hansenii*, and *Agrobacterium*, as well as algal species such as Rhizoclonium, Cladophora, Chaetomorpha, and Microdiction, have been evaluated [67,68,69]. BNC is a source of very pure cellulose (≥98%) because it does not involve pectin, hemicellulose, or lignin. Therefore, BNC is more elastic, thermally stable, and crystalline than nanofibers [68]. Furthermore, the BNCs possess a number of favorable characteristics such as porosity, moldability, average molecular weight, hemocompatibility, and mechanical stability [70]. The features and structure of BNC could be adjusted by changing the growth conditions such as bacterial strain type, incubation time, oxygen ratio, growing in a bioreactor and nutrient source [71].

**Figure 1:**
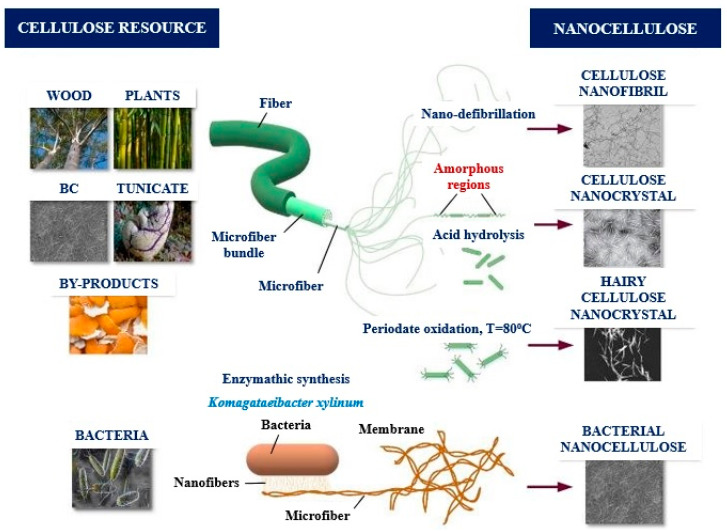
The various types of nanocellulose (reproduced from Negro et al. [77]).

Apart from the types of nanocellulose mentioned above, Van de Ven and Sheikhi [72] stated another nanocellulose type named hairy cellulose nanocrystalloid (HCNC) having some protruded cellulose part from both the ends of the crystalline body in some newsworthy studies. They are obtained from cellulose by any chemical treatments including periodate oxidation which bear not only crystalline but also amorphous regions [72]. Hairy cellulose nanocrystalloid offers conspicuously good physico-chemical features to be used in different applications such as cellulose hydrogel, heavy metal ion scavengers, super hydrophobic films, dye removal, food packaging and polymer reinforcement [73,74]. The various types of nanocellulose are depicted in Figure 1.

Nanocelluloses have carved out a niche in every sphere of life and they have gradually become the most attractive materials for the design of novel bio-based products. This ever-increasing interest is attributed to their outstanding characteristics. On the other hand, because of their hydrophilic structure which is attributed to the high content of hydroxyl groups, nanocelluloses have an aggregation problem in many non-polar solvents [6,75]. Thanks to the presence of functional groups in their structure, nanocelluloses are amenable to surface modification via different chemicals. These chemical modifications are necessary to regulate the interfacial features of nanocelluloses or to equilibrate their hydrophilic and hydrophobic conditions [6]. In the literature, there have been various processes such as non-covalent surface modification, amidation, oxidation, sulfonation, esterification, silylation, carbonylation (carbamyation, urethanization), etherification, polymer grafting onto, polymer grafting from, and click chemistry for chemical surface modification of nanocelluloses [6,64,76].

## 3. Nanolignin

Nanoparticles converted from lignin by various synthesis methods have a more developed surface area and can be modified to more accessible functional groups, which are important characteristics for high-value applications [78]. Therefore, lignin nanoparticles (LNPs) show enhanced or more different properties than starting lignins [19,79,80]. Therefore, the LNPs can be usable in different areas, including composite manufacturing, food packaging, and medicine, has attracted the interest of researchers due to their renewability, antioxidant activity, and thermal stability [25,78,81,82].

### 3.1. Synthesis Methods of LNPs

The production of LNPs includes a combination of physical and chemical techniques applying different lignin sources, such as kraft, alkaline, and organosolv lignins obtained from different raw materials (softwood, hardwood, or annual plant). In LNPs production, it can be listed as acid precipitation, self-assembly, mechanical applications (ultrasonication and homogenization), and other methods (chemical modification, microbial and enzyme-mediated, aerosol process, etc.) [25,78,79,83].

#### 3.1.1. Acid Shifting

This method is one of the most easily applicable methods and relies on the fundamental principle of altering the pH of lignin macromolecules dissolved in a solution. Frangville et al. [84] in their study, dissolved lignin in two different solutions. They used ethylene glycol and 1 M sodium hydroxide (NaOH) as solutions for the production of LNPs. Then they acidified the solutions with hydrochloric acid and nitric acid, respectively, to reach a low pH degree. During the dialysis of the ethylene glycol solution, they did not apply any extra treatments to the alkali solution. Rahman et al. conducted a study wherein they observed that nanoparticles synthesized through the utilization of ethylene glycol and castor oil exhibit a spherical morphology and measure between 15 to 20 nm in diameter [85]. Although the acid treatment used in this method is dangerous, it provides very good LNP production in terms of shape and stability [79].

#### 3.1.2. Self-Assembly Methods

These methods are the most common method to produce LNPs. The main goals of these methods are to produce nanoparticles by taking advantage of lignin solubility differences in different solvents. The approach has advantages, including that it is a simple process and uses a relatively minimal level of chemicals [79]. Organic solvents having high lignin solubility, such as THF, and DMF, are often utilized in these processes. After dissolved lignin in solvents, the solution is gradually introduced into an antisolvent, typically water, in order to generate the LNP [17]. In solvent precipitation method, the morphological properties of LNPs are affected by the miscibility of solvents. Well-mixed solvents form LNP more uniformly and in small diameters. However, there are disadvantages such as expensive solvents are difficult to recycle and some are toxic. The dissolved lignin is immersed in dialysis bags for the solvent exchange technique, and the LNPs are generated during dialysis. In the solvent exchange method, difficulty in controlling morphology and the low yield of LNPs production are the most important disadvantages [79].

#### 3.1.3. Mechanical Methods of LNPs Production

Ultrasonication and high shear homogenization play an essential part in the mechanical production of LNPs [86]. They are mechanical processes used to avoid or reduce the environmental concerns associated with solvents or other chemicals [17]. In these methods, no significant difference is observed in the original structure and functional groups of lignin. In addition, they have important advantages such as easy application and high controllability [79]. However, the LNPs created by the ultrasonication process are not uniform in terms of size or particle size distribution, which are dependent on the ultrasonic settings [86,87]. High shear homogenization, such as ultrasonication, is another effective method that can be used as a relatively simple and direct mechanical process for the formation of lignin nanoparticles [88].

#### 3.1.4. Other Preparation Methods

These techniques include enzymatic hydrolysis, interfacial crosslinking/polymerization, aerosol process, spray freezing, and electrospinning [17,78,79,89].

### 3.2. LNPs Characterization

The characterization of lignin nanoparticles is carried out in the literature studies by identifying different properties of lignin that are important for many areas of use, such as physical dimensions and morphology, colloidal behaviors, thermal and antioxidant properties, rather than structural diversity and heterogeneity [83].

Dimensional distributions of lignin nanoparticles are performed with scanning electron microscope (SEM), through-electron microscope (TEM) and atomic force microscope (AFM). In addition, it is possible to perform it with different measurement methods such as refractive index and sonic dismembranator [79]. Particle sizes of normal kraft lignin range from 10–100 μm [78]. Lignin nanoparticles generally vary according to their production method, but they can be produced in a wide range from 0.57 nm to 8000 nm [89].

As the particle size is reduced to the nanoscale, specific thermal characteristics such as melting, crystallization, and glass transition exhibit distinct alterations. The thermal characterization of nanocomposite materials is commonly achieved through the utilization of various analytical techniques such as Differential Scanning Calorimetry (DSC), Thermogravimetric Analysis (TGA), Thermal Mechanical Analysis (TMA), and Dynamic Mechanical Thermal Analysis (DMTA) [79]. Furthermore, zeta potential from colloidal solution properties, molecular weights of the obtained structures, and X-ray diffraction methods are widely preferred in the characterization of lignin nanoparticles [83,90].

### 3.3. LNPs Applications

The incorporation of nanofillers has been observed to enhance the mechanical characteristics, fire retardancy, stiffness, and thermal stability of polymers. Lignin nanoparticles are used in polymer matrices and nanocomposites for reinforcing these purposes with renewability, degradability, low cost, and low-density properties [91]. In addition, lignin possesses effective UV protection and photostabilization properties against UV radiation owing to the presence of phenolic, ketone, and other chromophore groups [92,93]. Therefore, lignin nanoparticles have the potential to be used in areas such as cosmetics, textile, and food package production, together with being non-toxic for humans [21]. Furthermore, the utilization of lignin nanoparticles has the potential to reduce pollution of the environment due to their fundamental properties including antibacterial activity, biodegradability, and reproducibility [17].

In recent years, many efforts have been made to utilize lignin nanoparticles for various purposes. Although it is commonly used as an additive, matrix material, filler, or reinforcing compound, antioxidant agent, and UV absorbent in composites, research continues in a variety of disciplines, including binders, dispersants, batteries, drug transport, heavy metal ion removal, paint removal, anticorrosion additive, and biosensors [17,21,25,78,94].

## 4. Lignocellulosic Material Based Sensors

### 4.1. Cellulose Based Sensors

Our world is surrounded by electronic devices which have key roles in our daily lives. Most of these electronic devices such as portable computers, digital cameras, mobile phones, tablets, electronic watches, and Bluetooth speakers are equipped with sensors. Sensors are analytical instruments that respond to different stimulating factors and are situated to recognize external factors for example light, motion, humidity, temperature, sound, or chemicals [95]. Sensors have been categorized by the International Union of Pure and Applied Chemistry (IUPAC) into three groups: physical sensors, chemical sensors and biosensors [96]. Sensor technologies have attracted significant notice in the application of different areas such as health, environment, and industry [97]. Notably, while people are using these technological devices they could choose the user-friendly, economical, sensitive, and excellently error-free detection tools [98,99,100].

“Green electronics” which are fabricated from natural materials, especially cellulose and its derivatives came into prominence in order to obtain the sustainability of electronics. Cellulose represents the total annual biomass production which is about 1.5 trillion tons, which is considered as an almost endless source of raw material for the increasing request for environmentally friendly and biocompatible products [101]. Nanocellulose-based materials with excellent electrical, optical, and mechanical properties have become an alternative to their commercial counterparts in various sensing applications such as environmental monitoring, food safety, physical sensing, human disease detection, and healthcare.

With the use of nanomaterials with advanced physicochemical properties, breakthrough developments have occurred in sensor technology in the last decade. However, due to their toxic effects on the environment and human health, research efforts have been focused on those types of nanomaterials generated from biomaterials [102,103,104]. Nowadays, the development of sensors based on nanomaterials especially nanocellulose has attracted enormous attention in the biomedical area for monitoring and managing human health [105,106,107]. It should be noted that nanocellulose-based materials generally exhibit excellent mechanical, thermal, chemical, physical, and barrier properties and they are chemically inert [108]. Concerning the considered applications, different modifications are available for the surface of nanocellulose supported with hydroxyl groups by introducing special functional groups.

#### 4.1.1. Cellulose-Based Physical and Chemical Sensors

Physical sensors are devices that can detect changes in physical stimulants and transform them into electrical signals [109]. Physical and chemical sensors are very important for environmental detection, and medical and industrial monitoring [110,111,112]. Recently, polymer composites are being used mostly in the fabrication of chemical sensors. The efficiency of chemical sensors is based on the transformation of chemical data into the signals which have been sent out. The chemical data could be the total composition of the analysis or the concentration of an analyte [113].

Nanocellulose has great potential with several interesting characteristics and genuine physical properties such as tensile strength, optical and electrical characteristics which make it a superior material for the production of chemical sensors [114]. Several studies have been guided on the use of nanocellulose in the production of physical and chemical sensors, some are described below.

##### Pressure/Strain Sensors

Strain sensors are the most important electrical sensors utilized for the measurement of mechanical quantities. These sensors convert mechanical deformation into a change in electrical resistance, which can then be measured [110]. Although it does not indicate a sensing ability to detect mechanical stimulants, the nanocellulose draws attention to its frequent use in pressure and strain sensors [115].

A multi-branched crystalline nanocellulose (CNC) was employed as a template for a strain sensor that prevents polyaniline (PANi) from aggregating while serving as a dynamic bridge and hydrogen bonding produced by Song et al. [116]. The combination of CNC-PANi with polyvinyl alcohol (PVA) and borax, hydrogen, and dynamic borax bonds a sensor was produced as the final sensor which showed repeatable sensitivity, uniform, and advanced self-betterment characteristics. The sensor could show a self-betterment characteristic up to 99.56% efficiency in 2 min. This product defined breaking strength as 171.52 KPa and 1085% stretchability.

Zhang et al. [117] improved a conductive hydrogel as a strain sensor which is highly sensitive by mixing the conductive materials with nanocellulose. Yan et al. [115] obtained nanocomposite hydrogel which showed excellent breaking strength (759 KPa), stretchability (974%), self-betterment (within 30 min) and self-adhesion as well as remarkable electrical conductivity (resistivity of 0.5 Ωm).

Su et al. [118], with inspiration from animal muscle, developed a dual network hydrogel by integrating graphene oxide (GO) into nanocellulose. The eventuating binary network hydrogel increased the average elongation at the break of the nanocellulose network from 86.2% to 748.0%. Moreover, the average tensile strength also significantly increases by 228.6%, as compared with the poly(AAm-co-AAc) hydrogels. The healing performance of the cut hydrogels are able to quickly renovate to 85.0% after 600 s of self-betterment.

##### Proximity Sensors

A non-contact sensor that detects the presence of an object when the target enters the sensor’s field is named a proximity sensor. Proximity sensors are used in several areas such as recycling plants, self-driving cars, mobile phones, assembly lines, and anti-aircraft systems. Nowadays, the technology returns to green fabricating integrated short-range proximity sensors to identify bio-signals without physical contact with different proximity sensing mechanisms [119].

**Figure 2:**
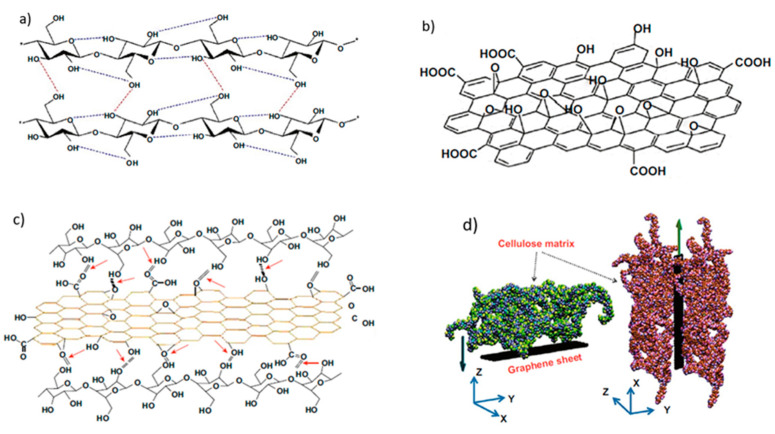
(**a**) Nanocellulose intra-intermolecular hydrogen bonding networks. (**b**) Graphene oxide proposed a structural model. (**c**) Schematic illustration of the hydrogen bonding (red rows) of regenerated graphene oxide nanosheets and nanocellulose molecular chains. (**d**) Model of the interfacial interactions between the graphene sheet and cellulose chain matrix (Reproduced from Brakat et al. [119]).

Thanks to its robust chain and homomolecular shape, the nanocellulose is very compatible to be used as a strong carrier or matrix for multifunctional nanocomposites. Figure 2 illustrates the strain sensor which is composed of nanocellulose chain matrix and highly conductive graphene sheets.

Sadasivuni et al. [120] have developed a transparent and flexible cellulose nanocrystal-reduced graphene oxide (CNCs-rGO) film as a proximity sensor for human finger detection and human skin identification by resistance change [121]. They have improved a CNC/GO-based proximity sensor by using an isophorone diisocyanate (IPDI) reagent and a standard reduction process and then carried out appropriate cooperation between detection capacity and fabrication cost. According to their results, the sensitivity of the m-r(CNC/GO) sensor alignment is about 5 times higher in amplitude compared to m-rGO because of its high surface-to-volume ratio and charge-storage capacity at junctions. This result belongs to the hydrophilic functional groups such as carboxyl groups which affect the peripheral humidity [122].

Also, the results show that the thermal degradation behavior of m-(CNC/GO) is higher than CNC and m-GO. The transmittance at 540 nm for both m-rGO and m-r(CNC/GO) showed a reduction from 85% to 30%. For the same number of sprayed layers m-r(CNC/GO) showed a better transmittance of 85% which decreased to 65% as the number of sprayed layers varied from 0 to 40. This result was related to the improved optical properties of m-r(CNC/GO) because of the transparent CNC. Interestingly, both CNC and rGO’s synergistic effects ensure the sensors’ better performance than the control sensor that made up rGO without CNC [113].

##### Temperature Sensors

Temperature sensors are the instruments which provide temperature measurement via an electrical signal [110]. Temperature mapping is essentially important for medical disciplines such as biotechnological applications, biological operations, etc. [113,123].

Yuen et al. [107] verified ultrathin bacterial cellulose (BC) sheets to compose flexible printed cycle boards which could be located on a human hand to follow up temperature and heart rate on target, showing advanced improvement in wearable medical sensing devices. The nanocellulose printed circuit boards could be produced via bot-tom-up and solution-based processing techniques.

Zhou et al. [124] designed a reduced graphene oxide(rGO)/bacterial cellulose (BC) aerogel film that demonstrates high anisotropy of the thermal conductivity and simply applicable potential in heat management in on-skin electronics.

Jung et al. [125] worked on a CNF-based thermoelectric sensor that was made by printing thermoelectric materials such as poly3,4 ethylenedioxythiophene/polystyrenesulfonate (PEDOT/PSS) on CNF films with inkjet printing. The study showed that the final product can detect a temperature change of 125 K with a sensitivity of 11 μV/K.

##### Humidity Sensors

Humidity sensors are generally made from polymers, metal oxides, nanomaterials, ceramics, and composites and they are important for human life and medical monitoring [126]. For the improvement of humidity sensors nanocellulose and its derivatives can be used in combination with the other materials. For the development of electronic skin and personal healthcare products; conformable and stretchable humidity sensors are considerable [109]. Moreover; the composites which contain nanocellulose and its derivatives can be a good substrate for sensing applications [113].

**Figure 3:**
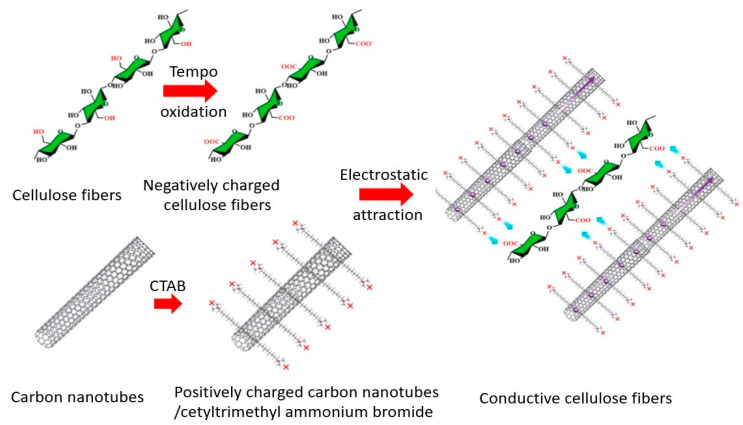
Schematic of the steps of TOCNF/CNT nanocomposite film formation (Reproduced from Zhu et al. [129]).

Zhu et al. [127] studied 2,2,6,6-tetramethylpiperidine-N-oxyl (TEMPO)-oxidized nanofibrillated celluose (TONC) and carbon nanotube (CNT) based highly sensitive and flexible humidity sensors which are very capable of monitoring human breath. Ayissi Eyebe et al. [128] utilized TEMPO-oxidized cellulose nanofibers (TONC) films as nonconductor material for humidity sensing because the dielectric constant of TONC films increases with humidity. Kafy et al. [121] have worked on a humidity sensor with CNC/GO composite film which is renewable and flexible with the help of the functionality of both crystalline cellulose and graphene oxide. Further, Zhu et al. [129] set up a humidity sensor that has a working principle based on the adsorption of positively charged CNT on the negative-ly-charged TONC, as shown in Figure 3.

In one of the most recent studies, Ginja et al. [130] exhibited the improvement of a humidity sensor predicated on a bacterial nano-cellulose (BNC) membrane which has been procured from Komagataeibacter xylinus. The mechanical and electrical properties of the membrane have been changed by the BNC because of its porous conformation. As a result, the membrane sensor could recognize the capacitance of the BNC sensor increases by 492 nF for an increase of 1% in the relative humidity.

##### Gas Sensors

The emissions of toxic products such as nitrogen oxides (NOx), carbon oxides (Cox), sulfur oxides (Sox), and ammonia (NH3) have recently increased considerably. Therefore, chemical sensing devices have been mainly developed for these chemicals which are harmful to all the world’s health and environment. Developments and recoveries of current chemical sensors and the improvement of new sensors via higher sensing performance and sensitivity with lower costs are needed to continue in various applications [131].

Núñez-Carmona et al. [132] have produced biologically consistent BC/ZnO gas sensors using BC (bacterial cellulose) as a substrate. As a result, good responses to nitrogen dioxide, acetone, and ethanol have been recorded. Liu et al. [133] fabricated p-type NiO nanoparticles with BC. In this study, for the determination of volatile organic compounds such as chlorobenzene, toluene, etc. NiO nanoparticles were converted to a sensor using BC.

Koga et al. [134] recommended a paper-based molecular sensor device with pencil-drawn graphite electrodes for NO_2_ sensing. The expendable sensor device was set up from a CNF paper substrate, a zinc oxide nanowire sensor, and a graphite electrode. The ZnO nanowires/CNF composite networks on the CNF paper show good adhesion. The resulting sensor showed an electrical resistance increase upon being subjected to NO_2_ with good sensitivity. Similar conclusions were reported by Pang et al. [135] for sensing NH_3_ vapors using CNF composite with polyaniline and TiO_2_. The polymerization of aniline on the TiO_2_/CNF surface forms a P–N junction. The polyaniline adsorbs NH_3_ gas molecules and the resistance of the composite material increases [136].

#### 4.1.2. Biosensors

Biosensors are devices that are being used to control ideal analyte materials in biological reactions and have sensing features. According to the IUPAC definition, biosensors are “chemical sensors in which the recognition system uses a biochemical mechanism” [137]. Biosensors can be classified on different basis based on the type of material analyzed or the transduction mechanism used by the sensor. Biosensors can be classified as optical, electrochemical, piezoelectric, electrical, pyroelectric, and gravimetric according to the transduction mechanism. Furthermore, the electrochemical biosensor can be divided into, amperometric, potentiometric, conductometric, impedimetric, and voltammetric. Furthermore, they can be classified as glucose, enzyme, cholesterol, urea biosensors, etc., according to analyte type [138].

**Figure 4:**
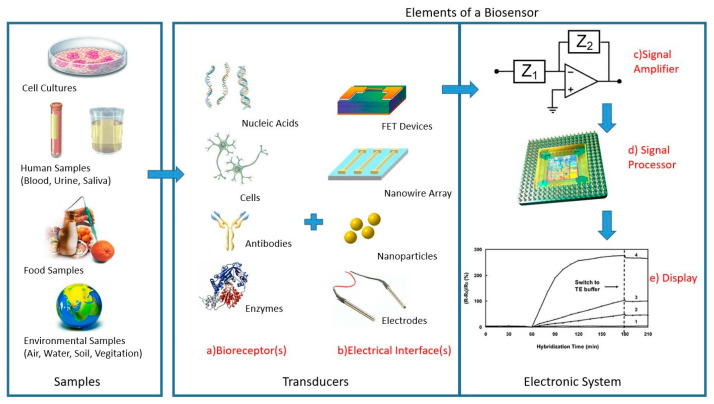
Elements and selected components of a typical biosensor (Reproduced from Grieshaber et al. [140]).

Biosensors are analytical devices that are capable of defining specific analytes using biological molecules and converting them into measurable signals using several kinds of technical mechanisms such as thermal, optical, or electrochemical, etc. Biosensors are making enormous research progress in various implementations such as agricultural, biomedical, and environmental. Alternative techniques and mechanisms were developed for the development of biosensors. The sensitive, selective, and biocompatible approach of the biosensors is according to the high affinity of the biorecognition element to form the complex with the specific analyte. This principle guides the innovation of a powerful analytical device that uses a bioreceptor molecule as a sensing element [139]. The elements of a biosensor are shown in Figure 4.

##### Glucose Sensor

Nanocellulose (NC) is one of the most appealing cellulose-based nanomaterials used in biosensors and biomaterials applications.

Neubauerova et al. [141] studied the colorimetric-based biosensor using nanocellulose-based supports for glucose detection in point-of-care testing. With this design, microcrystalline cellulose (MCC) samples were oxidized with TEMPO, sodium hypochlorite, and potassium bromide to have carboxylated nanocellulose. The NC-based biosensor was used to detect glucose in the urine samples of diabetes. The sensor was created by structuring GOx on the carboxyl-NC/cellulose substrate. The test strip was calibrated by incubating it in different concentrations of glucose. According to the result, 1.5 to 13.0 mM linear response was recorded for glucose. Furthermore, it was observed that the modified NC performed better color distribution and improved the process of glucose detection in terms of analytical performance.

Mun et al. [142] combined a conductometric glucose biosensor with a hybrid polymeric film composed of cellulose and zinc oxide (ZnO) nanoparticles. The actual level of the glucose biosensor increased with the growth of ZnO’s weight ratio. This improvement of the actual level might be based on its surface morphology and enhanced crystallinity of ZnO in the cellulose ZnO hybrid film (CZHF). The glucose biosensor was determined to be linearly sensitive to the glucose concentration up to 12 mM.

**Figure 5:**
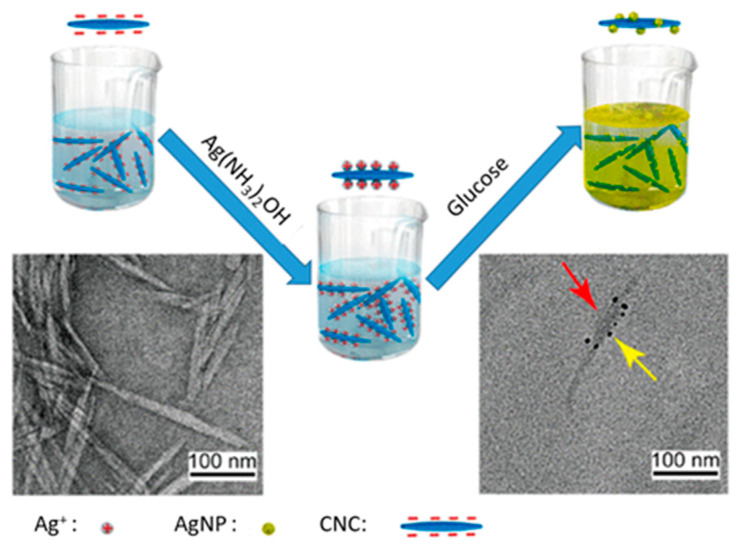
CNC-assisted generation of AgNPs via the redox reaction between glucose and silver ions (reproduced from Wang et al. [143]).

An example of using CNC as a substrate for the synthesis of silver nanoparticles (AgNPs) was given by Wang et al. [143]. The CNC has supported the production of AgNPs via in-situ reduction of Ag+ by glucose and is used for glucose sensing with high sensitivity. The resultant AgNPs have a lower minimal inhibitory concentration (MIC) than commercial ones because of the good dispersion with the presence of CNC [113]. The AgNPs/CNC combination was implemented for the improvement of a visual, quantitative, nonenzymatic, and high-sensitive assay for glucose detection in serum as shown in Figure 5. This trial is also beneficial for monitoring the concentration change of glucose in cell culture.

##### Enzyme Sensors

Protease biomarkers have recently received spectacular attention for wound monitoring. Proteolytic enzymes including serine proteases such as human neutrophil elastase (HNE) are model biomarkers because they successfully reflect the progress of wound healing [144,145]. Serine proteases are central to the pathology in a wide range of diseases including chronic wounds, cystic fibrosis, and acute respiratory distress syndrome [146].

Ling et al. [146] worked on a nanocellulose-based colorimetric biosensor for the detection of HNE with a peptine/cellulose combination that contains cotton-derived CNC. Results showed that TEMPO-oxidated nanocelluloses are ideal materials to be assembled into protease biosensors which showed potential for wound dressing as well as for an in-situ diagnostic point-of-care assessment for HNE in inflammatory diseases.

Fontenot et al. [147] developed a fluorescent sensor for sensing HNE with peptide-conjugated cellulose and nanocellulose. CNC peptide products show a higher sensitivity than other equivalents because of the larger specific surface area of the CNC films [113].

The analytes such as esterase enzyme, which have importance in biology and biotechnology, cellulose-based biosensors have been used for the detection. Derikvand et al. [148] performed quantitative imaging using a highly sensitive fluorescence scanner and demonstrated the effectiveness of the resulting paper-based esterase sensors. To represent the extensive applicability of this interference to cellulose bioactivation beyond traditional paper supports, they have prepared esterase sensors from cotton gauze purchased from a local pharmacy and BC produced by culturing Acetobacter xylinum.

##### Cholesterol Sensors

Cholesterol is a sterol that is synthesized in the liver and has a limit that should not be exceeded in people with cardiovascular disease. It is also an important part of the synthesis of some organic molecules such as vitamin D, steroid hormones, and bile acids. The screening of cholesterol relies mostly on the spectroscopies methods but nearly all the techniques are usually complex, and take too much time and cost.

For the detection of cholesterol, a new electrochemical biosensor was fabricated by Abdi et al. [149]. They have studied the immobilization of cholesterol oxidase (ChOx) on the polyaniline/crystalline nanocellulose/ionic liquid (IL) modified screen-printed electrode because CNC is a target component for conductivity and enzyme loading. The biosensor signalized a good detection limit (0.5 μM) and this device could be used to observe the cholesterol level between 0.001 and 12 mM while monitoring fast response and comparatively good consistency over 23 days.

In another study; a silylated graphene oxide-grafted chemically-modified nanocellulose (Si-GO-g-CMNC) was developed by Anirudhan et al. [150], for selective sensing of cholesterol. Different pulse voltammetry (DPV) analysis showed good linearity in the cholesterol sensing range of 0.6475–10.360 × 10^3^ μmol/L with 98.6 μmol/L limit of detection (LOD).

##### Urea Sensors

Nanocellulose-based biosensors have been proposed as rapid and applicable alternatives for the detection of urea [151,152]. Abdullahil et al. [153] developed a urea biosensor based on titanium oxide (TiO_2_)-cellulose composite. The results showed that the biosensor was highly sensitive to urea concentration below 10 mM and exhibited a wider detection range than the traditional biosensors because TiO_2_ can absorb a large amount of urease enzyme.

A film biosensor for the visual detection of urea has been fabricated successfully by Rehan et al. [154] with the combination of a pH-sensitive tricyanofuran-hydrazone dye into cellulose nanowhiskers [152]. The detection limit of the urea sensor was in the range of 50–1100 ppm which makes it suitable for food monitoring applications.

In another work, in the fabrication of a urea-sensing membrane by Dinh Doung et al. [155], the oxazine 170 perchlorate and ethyl cellulose (EC) were used as a matrix. This biosensor also was used to measure the concentrations of urea in the range of 0.01–0.1 M with a limit of detection (LOD) of 0.027 mM and 0.1–1.0 M with LOD of 0.224 mM. In addition to these favorable results, EC-based biosensors exhibited fast response time, high reversibility, and long-term stability.

##### Other Biosensors

In addition to the biosensors described above, some biosensor applications developed for various purposes are included in this section. Naghdi et al. [156] fabricated a curcumin-immersed bacterial cellulose (BC) nanopaper (CEBC) for optical sensing of human serum albumin (HSA). Some properties of BC nanopaper, such as it is transparent, flexible, porous, biodegradable, and printable, qualify it as an ideal platform. The CEBC was easily developed by soaking in curcumin solution and dried at 100 °C. Then, a nanopaper-based analytical device (NAD)-CEBC platform was prepared by printing a toner layer onto the dried CEBC film, creating hydrophobic walls with a lack of need for advanced instrumentation and using the minimum necessary sample volume (~5 µL) for HSA detection [157].

Abbasi-Moayed et al. [158] studied a ratiometric fluorescence (RF) sensor array for visual discrimination of biothiols. The product was manufactured on a BC nanopaper and it is able to discriminate among individual biothiols and their mixtures additionally the fast identification of biothiols in human plasma. Tian et al. [159] operated BC as a flexible surface-enhanced Raman spectroscopy (SERS) substrate which offers a useful platform for sensing implementations due to its porous structure resulting in remarkable SERS enhancement. Therefore, the researchers developed a 3D BC-based SERS substrate as a platform that can effectively detect and recognize bacteria *Escherichia coli*.

### 4.2. Hemicellulose Based Sensors

Hemicelluloses are biopolymers with multifunctional properties for biosensor utilization [160]. Hemicellulose has been reported to be used in drug delivery, tissue engineering, electronic skins (e-skins), human-machine interfaces, health monitoring, cancer chemotherapy, biosurfactant chemistry, metal ions films, hydrogels, conductive polymers, artificial intelligence applications, and dye adsorption.

#### 4.2.1. Hemicellulose Based Physical and Chemical Sensors

Due to its high amount of hydroxyl groups, hemicellulose is considered to be an ideal material for physical sensing (e.g., strain sensor). Hydroxyl groups can form cross-links through Van der Waals interactions or hydrogen bonding. as well as functionalized in terms of chemical cross-linking. Frequently, physical biosensors based on HP are added to the covalent bond of copolymer materials such as polyacrylic acid (PAA) or polyacrylamide (PAM), poly-N-isopropylacrylamide (PNIPAM), etc. together with the crosslinking element N,N′-methylenebisacrylamide. (MBA). By the way, composite hemicellulose biosensors offer more functions such as elongation, retention of water, freeze resistance, and adjustable distension behavior, which possess appealing nominees for sensing utilization [161].

Strain sensors that are both flexible, intelligent, and can be worn have received considerable attention for their adaptable uses in customized health checking, human-machine interaction, and electronic skin [162]. Zhang et al. [163] synthesized a multi-purpose hybrid hydrogel by utilizing natural polysaccharide hemicelluloses, polypyrrole (PPY), polyvinyl alcohol (PVA), tannic acid (TA) and polyacrylamide (PAM). Among these candidates, hemicelluloses were used as a blueprint for the synthesis of PPY polymerization in order to address the hydrophobicity issue and enhance conductivity. The permeability, mechanical features, bonding, and electrical conductance of the hydrogel were systematically assessed. Furthermore, the strain detection capabilities and sensibility performance of the hydrogel as a body-worn detecting device were also examined, leading to the discovery that the hybrid hydrogel exhibited exceptional and consistent adherence to diverse substrates, involving human skin tissue. A detector constructed using the hybrid hydrogel demonstrated a relative resistivity change of 1295% and a sensibility of 3.6% under a strain of 500%. The sensor effectively monitored pulse beats both at rest and during training, both of the strain signals associated with forefinger movement and elbow flexion. For this reason, the synthesis of the versatile hybrid hydrogel in this study holds promise as a body-worn detector for monitoring human movement, facilitating health-related diagnoses, and enabling electronic skin applications. The utilization of hemicelluloses in the preparation of hydrogels for biosensing purposes has been infrequently explored. However, in recent years, many researchers have investigated hemicellulose-based strain sensing applications.

Gong et al. [164] conducted a study where they prepared a hydrogel using polyacrylic acid (PAA) and hemicellulose nanoparticles derived from bleached bamboo kraft pulp. The hemicellulose nanoparticles were utilized as strengthening nanofillers at a weight percentage ranging from 0.1% to 0.5%. Subsequently, the hemicellulose nanoparticles were modified with tannic acid to create tannic acid-processed hemicellulose nanoparticles (TA@HC) for strain-detecting applications. The ionic hydrogels were synthesized by polymerizing acrylic acid in the presence of TA@HC nanofillers, followed by saturation with Aluminum (Al^3^⁺) ions. The resulting hydrogel exhibited a stretchability of up to 1060% and an induration of 1.52 MJ/m^3^. Additionally, the hydrogel, comprising polycyclic acid and tannic acid processed hemicellulose with Al^3^⁺ (PAA-TA@HC-Al⁺^3^) ions, possessed desired features such as anti-ultraviolet, anti-oxidative along with antibacterial features. Notably, the ionic hydrogel, when used as a wearable strain detector, exhibited high sensibility and demonstrated excellent performance in detecting various human movements, including weak pulse, breathing, and speech. To meet specific utilization requirements, natural hemicelluloses need to be processed and new derivatives, such as etherified and fluoridated hemicelluloses, must be synthesized.

**Figure 6:**
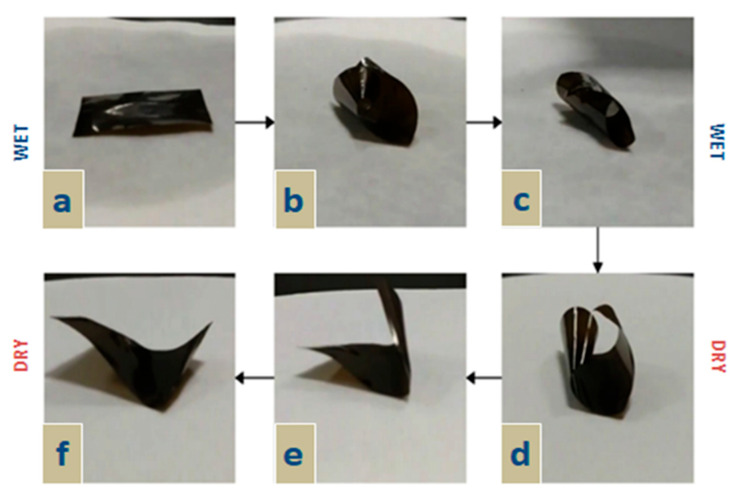
The morphology of the hybrid film changed at different humidity (**a**–**c**) exposed to humidity; (**d**–**f**) were exposed in dry conditions (reproduced from Rao et al. [165]).

Rao et al. [165] conducted a study where they utilized bamboo-derived hemicelluloses and subjected them to treatment with 2,3-epoxypropyltrimethyl ammonium chloride (ETA) in an alkaline environment [166]. Subsequently, the hemicelluloses were further modified through quaternary ammonium functionalization, resulting in the inclusion of amino groups and the manifestation of cationic and ampholytic properties in the molecular structure of hemicelluloses. The mentioned functional features enabled the incorporation of hemicelluloses into graphene oxide. In this research, homogeneous films were prepared using quantified hemicellulose (QH) and graphene oxide (GO), which led to improved actuator movement driven by moisture gradient. The curling movement of the film at different moisture levels is depicted in Figure 6.

The hybrid film exhibited rapid upward tilting within seconds (Figure 6b) and reached maximum tortuosity under moist conditions (Figure 6c). Subsequently, the hybrid film initiated the restoration of its initial state. (Figure 6d,e), and the extent of recovery attained its peak in the dry position (Figure 6f). This observation demonstrated the excessive sensitivity of the hybrid film to moisture. This sensitivity was ascribed to the special structure of the film, which activated the actuator to respond excessively to humidity. The hybrid film contained countless oxygen-containing functional groups (-COO- and -OH), which facilitated its easy absorption of moisture. The water molecules acted as crosslinking agents, forming hydrogen bonds with GO sheets and QH chains, resulting in unequal distribution of internal tensions. Consequently, the configuration of the hybrid film changed accordingly. These results suggested that this hybrid film can be utilized in humidity sensors or water level switches.

In recent research, laser-induced graphene has been developed using xylan, a plentiful and eco-friendly biopolymer, and has shown promising applications in temperature sensing. For instance, Kulyk et al. [167] aimed to demonstrate the utilization of laser-induced graphene (LIG) derived from xylan, which is a readily available and often underutilized biopolymer, for temperature sensing purposes. They developed a temperature sensor by utilizing Laser-Induced Graphene (LIG) derived from modified xylan. The process involved irradiating a 6 × 12 mm^2^ section of the xylan film that was affixed to filter paper, employing a power of 1.5 W, a scan speed of 30 mm s⁻^1^, and a line separation of 0.1 mm. Tin-coated 20 AWG copper wires were connected to each end of the detector using silver paste. The researchers successfully demonstrated that LIG can be produced by irradiation of fire-inhibited xylan film, which is a plentiful and eco-friendly biopolymer. This resulting material was utilized as a proof-of-concept temperature detector, exhibiting a sensibility of 1.29 Ω ℃⁻^1^. In general, this research enhances our comprehension of the process of synthesizing laser-induced graphene and broadens the scope of predecessor substrates and resulting materials in this particular field.

Chemical sensors have a crucial role in surrounding control and provide valuable information regarding industrial manufacturing processes, quality control of food and beverages, and various other utilization Chemical sensors are devices operating on electrochemical principles used for surveillance and monitoring, capable of interacting with a wide range of chemical components and providing valuable insights. In addition to high accessibility, bio-compatibility, renewable structure, and biodegradability, hemicellulose possesses unique material properties such as dimensional stability and a poor thermal dilatation coefficient. These characteristics make hemicellulose an excellent material for chemical sensing applications [168].

The wastewater discharged by industries such as leather, textiles, printing, and others contain toxic substances, including refractory dyes, which constitute a risk to the water quality of oceans and the organisms living in them [169]. Methylene blue is a commonly used dye in the aforementioned industries. This methylene blue dye is typically present in watery solutions as a cation, and the main mechanism for the adsorption of ionic dyes is electrostatic attraction. Hemicellulose, with its anionic properties, exhibits excellent absorbent properties, making it a suitable material for methylene blue absorption. Additionally, hemicellulose possesses antioxidant and non-toxic properties, making it a promising biomass material for the removal of organic dyes. For example, Seera et al. [170] investigated the synthesis and characterization of a xylan-gelatin cross-linked hydrogel with the ability to adsorb methylene blue. The composition of hemicellulose and the network structure of the hydrogel are crucial factors that influence the adsorption capacity of the hemicellulose hydrogel. However, it is important to note that hemicellulose is susceptible to hydrolysis in acidic environments, limiting its use in highly acidic conditions [171].

Recent research has highlighted the promising adsorption capabilities of hemicellulose-based hydrogels for metal ions such as Pb^2^⁺, Cu^2^⁺, Cd^2^⁺, and Zn^2^⁺. In research by Kundu et al. [172], a hydrogel blend consisting of xylan and β-cyclodextrin (βCD) was formulated, and it demonstrated effective absorption of Ni^2^⁺ and Cd^2^⁺ ions. During the hydrogel synthesis ethylene glycol glyceride was employed as a cross-linker agent. The hydrogels exhibited superior adsorption performance for Cd^2^⁺ compared to Ni^2^⁺. This improvement in absorption ability was attributed to the enrichment of hydroxyl groups in the hydrogels. Studies demonstrated heavy metal ions and hydroxyl groups have strong proximity. Another strategy to enhance the adsorption capacity of hemicellulose hydrogels is by increasing the content of carboxyl groups, as research has indicated that carboxyl groups also contribute to improved adsorption capabilities in hydrogels.

#### 4.2.2. Hemicellulose Based Biosensors

**Figure 7:**
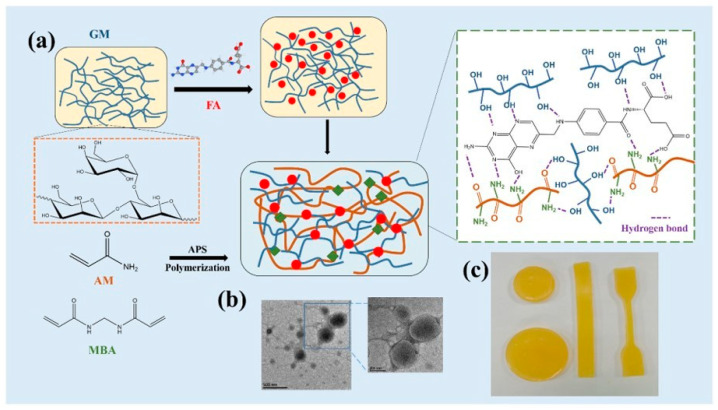
(**a**) Schematic illustration of the preparation process of GM hydrogels; (**b**) TEM images of the hybrid solution before heat-induced molding; (**c**) The GM hydrogels molded into various shapes (Reproduced from Ling et al. [161]).

Hemicellulose-based biosensors offer great potential as analytical probes for various analytical tasks. Several biosensors based on hemicellulose have been developed using different detection platforms, finding applications in pathogen detection and diagnostics. For instance, Ling et al. [161] fabricated innovative double network (DN) hydrogels utilizing galactomannan (GM) polysaccharide, as depicted in Figure 7. Hydrogen bond connections and covalent bond networks were established with the incorporation of folic acid (FA) and polyacrylamide (PAM), respectively. After 24 h of incubation, GM hydrogels exhibited more than 80% viability, indicating excellent antibacterial properties against *Escherichia coli*. The amount of FA fusion in GM polysaccharides was found to be crucial in achieving these antibacterial effects. Furthermore, the introduction of FA was shown to enhance the conductibility and antibacterial capacity of the GM hydrogels. Consequently, these multifunctional hydrogels hold significant potential as conductant sensors and anti-fatigue/bacterial agents, particularly in the field of wearables.

Polysaccharide-based carbon quantum dots (CQDs) have gained significant attention in the field of sensing, although numerous CQDs-based sensors primarily rely on their optical properties. In research by Han et al. [173], the selectivity and reducibility of xylan-based CQDs were considered, leading to the establishment of a highly delicate sensor for dopamine (DA) sensing using a greenly synthesized CQDs-based nanocomposite. Initially, the CQDs derived from xylan were utilized as both the reductor and stabilizer to efficiently reduce graphene oxide (GOx) and compound silver nanoparticles (AgNPs). Subsequently, the Ag@CQDs-GOx nanocomposite was synthesized and immobilized onto a glassy carbon electrode (GCE) to fabricate the detector. The electrochemical detection of DA holds significant importance in the research of diagnosis due to its potential feasibility in the treatment of neurochemical diseases. İn optimized conditions, for monitoring DA the sensor exhibited a linear range from 0.1 to 300 μM and it had a poor sensing limit of 1.59 nM. The sensor effectively detected DA in samples of dopamine hydrochloride infusion and bovine serum solution. This suggested approach has the capacity to broaden the utilization of carbon quantum dots (CQDs) based on polysaccharides. Moreover, the sensor itself provides a precise and responsive analytical platform for dopamine-related clinical diagnostics and drug screening.

Katrlík et al. [174] conducted a study on the fabrication of a biosensor using Surface Plasmon Resonance (SPR) technology. The biosensor was based on mannan extracted from Candida dubliniensis yeasts. The mannan was subjected to biotinylation and subsequently anchored onto an SPR chip modified with streptavidin, utilizing a polycarboxylate matrix. The analytical characteristics of the developed SPR biosensor were examined, and its interaction with Concanavalin A (Con A) was investigated. The biosensor was utilized to evaluate the levels of antibodies against mannan from C. dubliniensis in rabbit sera following immunization with mannan, mannan-albumin conjugate, and whole-cell preparations of inactivated yeast. The biosensor feedback displayed linearity up to 16 nM Con A lectin with 0.1 nM sensing limit and 2 min’ feedback time. Antimannan antibodies were quantified using ELISA on a microplate that was modified with Concanavalin A (Con A) and mannan. The half-life of the interplay between antisera and mannan using the SPR biosensor was determined, indicating that immunization with the mannan conjugate yielded the most efficient production of mannan-specific immunoglobulins with the highest binding affinity for mannan derived from Candida dubliniensis. This study presented an effective biosensing tool for investigating and understanding the proximity and interaction mechanisms of lectins and anti-carbohydrate antibodies, offering potential applications in the fields of immunology research, biotechnology, clinical diagnosis, treatment, and the study of diseases caused by pathogens.

### 4.3. Lignin Based Sensors

#### 4.3.1. Lignin Based Physical and Chemical Sensors

Recently, there has been an increasing series of studies highlighting the significant potential applications of lignin. Lignin has found applications in various fields such as sunscreen agents, drug distribution, functional fillers, abrasion protection, and energy storage. Its non-toxicity, biodegradability, good mechanical properties, and other qualities make lignin a popular raw material for physical sensors. Ongoing research focuses on the preparation of sensors with high lignin content and multifunctional properties.

Han et al. [175] fabricated a polyvinyl alcohol (PVA) hydrogel integrated with lignin-silver hybrid nanomaterials, demonstrating remarkable compressibility. The lignin-silver hybrid nanomaterials acted as strong modifiers of the hydrogel, providing powerful hydrogen bonds and facilitating electron shift. By harnessing these exceptional characteristics, the PVA/lignin-silver hybrid nanomaterial hydrogel holds great potential as a pressure-sensitive sensor for monitoring signals. The demethylation process of lignin led to the liberation of phenolic hydroxyl groups, which enhanced the adhesive properties of lignin and improved its reducibility [176].

Chen et al. [177] conducted a study where they applied biodegradable and renewable lignin material onto the surface of a metal electrode to create humidity-sensitive sensors. The sensing productivity for humidity detection of lignin-based quartz crystal microbalance (QCM) sensors were investigated using both symmetric and ringed electrode configurations, employing the swing circuit method. Based on the experimental findings, it was observed that the humidity sensitivity of the QCM sensor utilizing a ringed electrode configuration (61 Hz/%RH) was greater than that of the sensor employing a symmetric electrode configuration within the RH range of 11.3% to 97.3%. The underlying mechanism responsible for the improved sensitivity of the QCM humidity sensor, which is based on lignin and features a ringed electrode configuration, was examined and explained through the utilization of equivalent electronic circuit analysis and simulation methods. The findings of this study provide strong evidence supporting the suitability of lignin as a highly effective material for humidity detection. Furthermore, the optimization of the electrode structure configuration utilizing the fringing field effect emerges as a promising strategy for enhancing the humidity sensitivity of QCM sensors.

In this research, Sun et al. [178] presented a simple procedure for synthesizing lignin-based carbon dots (L-CDs). The raw materials used in the synthesis process included lignin, citric acid, and ethylenediamine. The researchers optimized the synthesis conditions to enhance the fluorescence lifetime of the L-CDs. Furthermore, the structure and pH-responsive characteristics of the lignin-based carbon dots were thoroughly examined in this study. By combining L-CDs, N-isopropylacrylamide (NIPAM), and polyvinyl alcohol (PVA), the researchers successfully synthesized fluorescent hydrogels that exhibited pH/temperature dual response through free radical polymerization. The diameter of the L-CDs ranged from 2 to 5 nm, and they exhibited a crystalline structure resembling graphene. Under the optimized conditions, the L-CDs exhibited a fluorescence lifetime of approximately 12 ns and a quantum yield of 43.9%. Within the pH range of 1 to 10, the fluorescence intensity of the L-CDs exhibited a proportional relationship with the pH value. Furthermore, researchers synthesized a pH/temperature dual-responsive hydrogel by incorporating L-CDs. The hydrogel demonstrated a higher value of elastic modulus (G′) in comparison to the viscous modulus (G″). They were also noted that the temperature sensibility and water retention rate of the hydrogel gradually declined as the PVA content exceeded 10 wt%.

Due to their favorable characteristics, lignin-based materials have garnered significant interest as sensing materials in recent studies. Lignin-based materials exhibit high thermal stability, making them suitable for applications that involve elevated temperatures. They also possess strong UV absorption capabilities, which can be advantageous in sensing applications that require protection from UV radiation. Additionally, lignin-based materials exhibit excellent water stability, maintaining their structural integrity even in humid or aqueous environments. These materials offer similar attractive morphological and mechanical qualities, such as flexibility and mechanical strength, which are desirable for sensor development. Furthermore, lignin-based materials are considered low-cost and sustainable, aligning with the growing demand for environmentally friendly and economically viable sensing solutions. In general, lignin-based materials show great potential for the advancement of innovative detecting materials with exceptional performance attributes.

In a study carried out by Joshi et al. [179], the synthesis of zinc oxide (ZnO) nanorods with a hierarchical-type structure was achieved using fragmented lignin. The researchers isolated lignin from cossene using a microwave-assisted procedure and fragmented it under alkaline conditions with the addition of hydrogen peroxide. Subsequently, the disjoined lignin was utilized as a pattern for synthesizing the zinc oxide (ZnO) nanorods. Powder X-ray diffraction (XRD) analysis was conducted to analyze the resulting ZnO samples, which revealed a hexagonal structure. In comparison to ZnO instances without disjoined lignin, the presence of disjoined lignin resulted in the formation of a self-assembled hierarchical nanostructure, comprising nanorods with lengths ranging from 200 to 500 nm and a diameter of 30 nm. The inclusion of disjoined lignin had a notable impact on the extent and morphology of the ZnO nanoparticles, ultimately giving rise to the observed hierarchical structure.

#### 4.3.2. Lignin Based Biosensors

Lignins have gained significant attention in several areas such as electrochemistry, pharmacy, sensors, and biomedicine, because of their versatile applications. Although the utilization of lignin as a biosensor for medical or bacterial sensing is still relatively uncommon, there are researchers actively exploring the potential of lignin-based biosensors.

**Figure 8:**
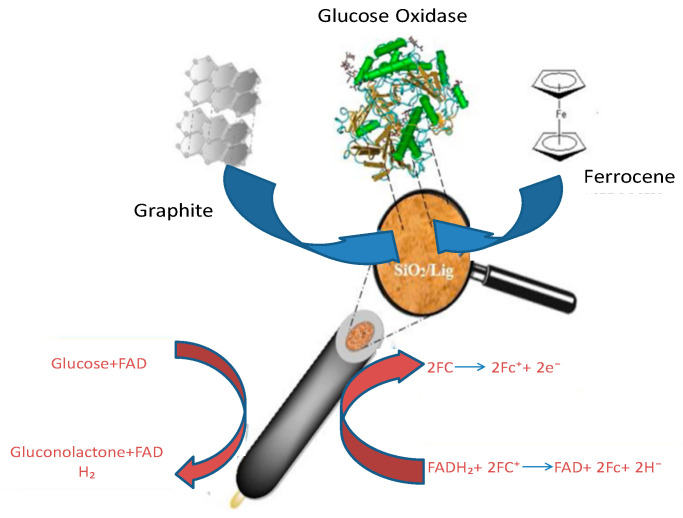
The acting of GOx-SiO2/Lig into CPE for β-d-glucose (Reproduced from Jędrzak et al. [180]).

Jędrzak et al. [180] conducted a study in which they introduced a new method for fabricating an enzyme biosensor utilizing an affordable and functional silica/lignin (SiO₂/Lig) hybrid material. The researchers utilized a functional biohybrid SiO₂/Lig material as a platform for immobilizing glucose oxidase (GOx) through absorption on that surface. Mechanisms involved in the immobilization process are illustrated in Figure 8.

The immobilized quantity of Gox in the SiO₂/Lig composite was 25.28 mg g⁻^1^, exhibiting twice the amount compared to its presence on non-functionalized SiO₂. The GOx-SiO₂/Lig system was integrated with single-walled carbon nanotubes and platinum nanoparticles as a supportive framework for the development of an AI-generated glucose biosensor. Additionally, the ferrocene redox-mediated GOx-SiO₂/Lig-based carbon paste electrode was assessed as an active ingredient in the second descendants’ glucose biosensor. The findings suggest that GOx-SiO₂/Lig could be the preferred material for developing an efficient and cost-effective biosensor that can be utilized in various electrode configurations.

**Figure 9:**
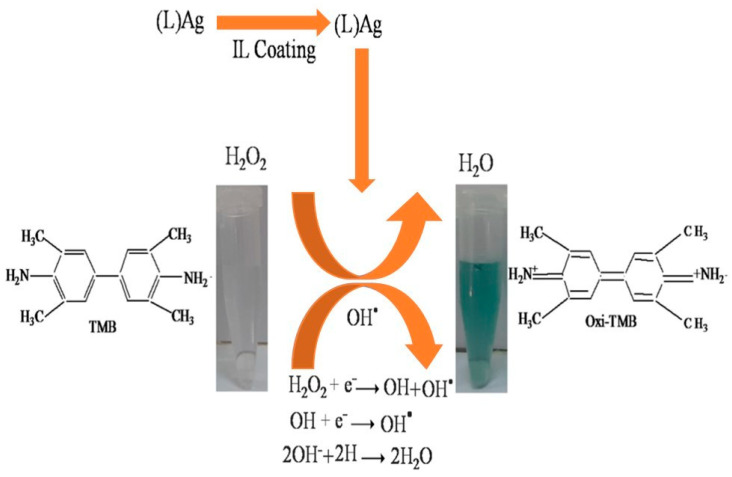
Proposed reaction mechanism for the detection of H_2_O_2_ (Reproduced from Nishan et al. [181]).

Nishan et al. [181] conducted a study where they utilized lignin as a stabilizer and reductive agent to prepare silver nanoparticles (AgNPs). The synthesized AgNPs were subjected to a coating process using an ionic liquid (1-H-3-methylimidazolium acetate) to produce ionic liquid-coated lignin stabilized silver nanoparticles (LAgNPs). The application of the coating resulted in improved catalytic activity, immobility, conductibility, and diffusibility of the nanoparticles, thereby enabling their effective utilization as peroxidase mimics for the colorimetric detection of hydrogen peroxide (H₂O₂). The reaction mechanisms involved in this process were depicted in Figure 9. The developed protocol involved the combination of ionic liquid-coated nanoparticles (IL-NPs) with a solution of 3,3′,5,5′-tetramethyl benzidine (TMB) and a wadding solution to create a sensor that detects hydrogen peroxide (H₂O₂) based on colorimetric principles. In optimized conditions, the sensor demonstrated excellent performance with a wide linear range (1 × 10⁻⁹–3.6 × 10⁻⁷ M), a low detection limit of 1.37 × 10⁻⁸ M, and a quantification limit of 4.59 × 10⁻⁸ M, with an R^2^ value of 0.999. The suggested sensing probe presents a straightforward, fast, high degree of sensitivity, selector, and stable biomimetic catalyst approach for colorimetric detection of hydrogen peroxide (H₂O₂), with potential applications in medical diagnostics. The sensor has demonstrated its selectivity in detecting hydrogen peroxide even in the existence of other concurrent substances. Furthermore, it has been effectively employed in the sensing of H₂O₂ in real examples.

Lastly, tuberculosis is a highly contagious disease caused by Mycobacterium tuberculosis, and its accurate and timely diagnosis remains a challenge. Current diagnostic methods are limited in their sensitivity and time-consuming nature. In a study by Tai et al. [182], a green graphene nanofiber laser biosensor (LSG-NF) decorated with synthetic silver nanoparticles (AgNPs) obtained from lignin extracted from palm oil was developed. To affirm the sensing capability, a selective DNA sample was captured on AgNPs and surveyed for specific bonding to Mycobacterium tuberculosis target DNA through selective hybridization and mismatch analysis. Successful immobilization and hybridization of DNA were confirmed through the identification of phosphorus and nitrogen signals using X-ray photoelectron spectroscopy (XPS) and Fourier transform infrared spectroscopy (FTIR) analyses were conducted. The analysis demonstrated good replicability and stability. This approximation presents a potential and cost-effective sensing system for the detection of Mycobacterium tuberculosis biomarkers, providing a novel avenue in medical diagnosis. By harnessing lignin as a key component in the synthesis of synthetic silver nanoparticles, this study emphasizes the capacity of lignin as a valuable material in advancing the field of biosensors for disease detection.

## 5. Conclusions

Due to their unique features and excellent properties (surface chemistry, high aspect ratio, high surface area, improved mechanical strength, thermal stability, flexibility, biodegradability, biocompatibility, nontoxicity, and renewability), the lignocellulosic bionanomaterials have great potential to be used in biosensor applications. The review has revealed that nanocellulose and nanolignin can be utilized as supporting material in biosensors. Although nanocellulose is nonconductive, its composites with conductive polymers and nanoparticles are electrically conductive and can be used in biosensor applications. In particular, cellulose- and lignin-based nanomaterials have unique chemical structures and they offer a good platform to accomplish the immobilization process of bioactive molecules in biosensors. These green biosensors are promising platforms since they are low-cost, portable, lightweight, suitable for miniaturization, and consumer-friendly, and thus meet the requirements for on-site detection. Due to ever increasing need for low-cost and eco-friendly materials, the demand for lignocellulosic bionanomaterials-based biosensors will increase considerably. Therefore, it is anticipated that future research efforts will focus on exploring the potential benefits of using lignocellulosic bionanomaterials-based biosensors for biodefense, environmental monitoring, healthcare, food safety, and biological and medical applications. There are a lot of examples of lignocellulosic bionanomaterials-based biosensors that have been developed for a broad range of applications at the laboratory scale. Nevertheless, only a few bio-based sensors are commercially available. Thus, more efforts are needed in research innovation toward the development of automated, real-time, and continuous biosensing devices for different applications.

## Data Availability

Data will be made available on request.
